# Authors’ response: Addressing the blind spot of self-reported adherence in the era of long-acting PrEP

**DOI:** 10.2807/1560-7917.ES.2026.31.2.2600010

**Published:** 2026-01-15

**Authors:** Davide Moschese, Andrea Antinori, Andrea Gori, Valentina Mazzotta, on behalf of the authors of the original article

**Affiliations:** 1 Department of Infectious Diseases, Luigi Sacco University Hospital, ASST Fatebenefratelli Sacco, Milan, Italy; 2 Regional AIDS Reference Center, National Institute for Infectious Diseases, Lazzaro Spallanzani IRCCS, Rome, Italy; 3 Health Direction, National Institute for Infectious Diseases, Lazzaro Spallanzani IRCCS, Rome, Italy; 4 Department of Biomedical and Clinical Sciences, Università degli Studi di Milano, Milan, Italy; 5 Centre for Multidisciplinary Research in Health Science (MACH) , Milan, Italy; 6 https://doi.org/10.2807/1560-7917.ES.2025.30.39.2500739

**Keywords:** HIV, pre-exposure prophylaxis, PrEP, long-acting injectable, cabotegravir, CAB-LA, adherence

**To the editor**: We thank Brunoro et al. for their thoughtful letter and for sharing their real-world experience in implementing HIV pre-exposure prophylaxis (PrEP) in Italy. We appreciate their support of our conclusions and their contribution to the ongoing discussion on optimising access to long-acting injectable cabotegravir (CAB-LA).

We agree that accurately assessing adherence to oral PrEP remains challenging. Pharmacy-based metrics, such as the proportion of days covered (PDC), can provide useful complementary information. However, several considerations are essential when interpreting these measures for CAB-LA prioritisation.

Firstly, pharmacy refill–based indicators such as PDC do not necessarily equate to biological protection from HIV. Oral PrEP effectiveness depends not only on pill possession but also on sexual exposure, dosing strategies (including event-driven use), and the pharmacokinetic forgiveness of tenofovir disoproxil fumarate/emtricitabine. Even with a daily PrEP schedule, taking at least four doses per week is considered sufficient for substantially reducing HIV acquisition risk [1,2], corresponding to a PDC lower than 60%. Consequently, a PDC below conventional thresholds (80%) does not necessarily identify persons who would unequivocally benefit more from long-acting formulations.

Secondly, interpretation of PDC values exceeding 100% warrants caution. While informal pill sharing is one possible explanation, alternative scenarios — including early refills, stockpiling after sexual abstinence, regimen changes or administrative factors — cannot be excluded. Furthermore, in the absence of a centralised system for recording PrEP prescriptions and supplies, some users may obtain the drug from multiple centres, including both clinical and community-based services. Attributing excess coverage primarily to medication sharing may therefore lead to overinterpretation and not fully capture the complexity of real-world PrEP use.

Thirdly, although CAB-LA offers clear advantages by ensuring appropriate drug administration in clinical settings, its role in mitigating informal medication sharing or redirecting individuals towards formal care pathways remains speculative. Structural and social determinants that drive informal PrEP use are unlikely to be fully addressed by formulation choice alone and require broader public health and service-level interventions.

In our implementation programme, CAB-LA eligibility was not based solely on perceived or measured oral PrEP adherence but on a comprehensive, person-centred clinical, social and behavioural assessment. Central to this approach was the establishment of a trusting clinician–user relationship, supported by shared decision-making and an open dialogue about HIV exposure risk, which is inherently dynamic and may vary across time and life circumstances [3]. Within this framework, self-reported adherence and behaviours were explored through targeted questionnaires, interpreted alongside clinical evaluation and structural factors. Moreover, tailored and targeted counselling about risk perception and use of PrEP was provided at each assessment to address cultural barriers associated with suboptimal adherence [4].

We agree that combining multiple sources of information can enrich clinical assessments. Pharmacy refill indicators or electronic prescribing tools may be valuable for estimating adherence patterns and informing the potential need for long-acting PrEP at a population level [5]. However, decisions regarding CAB-LA initiation should ultimately be made on a case-by-case basis, especially in settings with limited access to long-acting options, to avoid misclassifying individuals who may benefit from long-acting prevention strategies and to adhere to a cost-effectiveness rationale [6].

## References

[1] Landovitz RJ Tao L Yang J de Boer M Carter C Das M Type 1 human immunodeficiency virus (HIV-1) incidence, adherence, and drug resistance in individuals taking daily emtricitabine/tenofovir disoproxil fumarate for HIV-1 pre-exposure prophylaxis: pooled analysis from 72 global studies. Clin Infect Dis. 2024;79(5):1197-207. 10.1093/cid/ciae143 38484128 PMC7616831

[2] European AIDS Clinical Society (EACS). EACS Guidelines version 13.0. Brussels: EACS; 2025. Available from: https://www.eacsociety.org/guidelines/eacs-guidelines

[3] Zeballos D Guimarães NS Pereira M Magno L Dourado I. Performance of adherence measures for oral, tenofovir-based HIV pre-exposure prophylaxis: a systematic review. AIDS Behav. 2025;29(9):2841-54. 10.1007/s10461-025-04741-8 40327271

[4] Mazzotta V Caruso E Tavelli A Esvan R Lanini S Micheli G HIV oral pre-exposure prophylaxis effectiveness, adherence, and discontinuation in an Italian multicentric access program: ItaPrEP Study. Open Forum Infect Dis. 2025;12(9):ofaf539. 10.1093/ofid/ofaf539 40980566 PMC12448426

[5] Pyra M Rusie L Castro M Keglovitz Baker K McNulty M Bohm N A taxonomy of pragmatic measures of HIV preexposure prophylaxis use. AIDS. 2020;34(13):1951-7. 10.1097/QAD.0000000000002618 33009011 PMC7856306

[6] National Institute for Health and care Excellence (Nice). Cabotegravir for preventing HIV-1 in adults and young people; Technology appraisal guidance. Reference number:TA1106. Manchester: NICE. [Accessed: 2 Jan 2026]. Available from: https://www.nice.org.uk/guidance/ta1106 41570163

